# Catechin Reduces Blood Pressure in Spontaneously Hypertensive Rats through Modulation of Arachidonic Acid Metabolism

**DOI:** 10.3390/molecules27238432

**Published:** 2022-12-02

**Authors:** Fawzy Elbarbry, Gabriel Jones, Aimy Ung

**Affiliations:** School of Pharmacy, Pacific University Oregon, Hillsboro, OR 97123, USA

**Keywords:** catechin, captopril, SHR, arachidonic acid, metabolism, soluble epoxide hydrolase, hypertension, 20-HETE, EETs

## Abstract

**(1) Background**: hypertension affects approximately half of the adults in the United States (roughly 116 million). The cytochrome P450 (CYP)-mediated metabolism of arachidonic acid (AA) in the kidney has been found to play a major role in the pathogenesis of hypertension. This study examines the anti-hypertensive effect of the natural polyphenolic compound catechin (CAT) and investigates if it impacts the metabolism of AA in the kidney in comparison to captopril (CAP): a commonly used antihypertensive drug. **(2) Methods**: spontaneously hypertensive rats (SHR) were randomly divided into five groups. The treatment groups were administered CAT in drinking water at doses of 10 and 50 mg/kg. A positive control group received CAP at a dose of 10 mg/kg in the drinking water, and one group received both CAP and CAT at doses of 10 mg/kg and 50 mg/kg, respectively. Blood pressure was monitored weekly for five weeks. The activity of the two major enzymes involved in AA metabolism in the kidney, namely CYP4A and soluble epoxide hydrolase (sEH), were analyzed. **(3) Results**: CAP monotherapy was found to reduce blood pressure compared to the control untreated rats but did not demonstrate any effect on AA metabolism. Low- and high-dose CAT resisted the rise in blood pressure observed in the untreated SHR and significantly lowered blood pressure compared to the control group, respectively. Only rats treated with high CAT doses demonstrated significant inhibition of CYP4A and sEH enzyme activities. The coadministration of CAP and a high dose of CAT resulted in more pronounced blood pressure-lowering effects, but no more significant effects on AA metabolism were found compared to a high dose of CAT alone. **(4) Conclusion:** the modulation of AA metabolism in the kidney contributes, at least partially, to the blood pressure-lowering effect of CAT in SHR rats.

## 1. Introduction

Recent statistical studies indicate that cardiovascular disease (CVD) is the leading cause of death in the United States, causing one in every five deaths in 2020 [1]. Hypertension, defined as systolic blood pressure ≥130 or diastolic blood pressure ≥80, is the most predominant CVD and affects almost half of the adult US population [1]. Unfortunately, less than 25% of patients with hypertension have their blood pressure controlled [1,2]. Uncontrolled hypertension increases the risk of heart attack and stroke [1].

**Figure 1:**
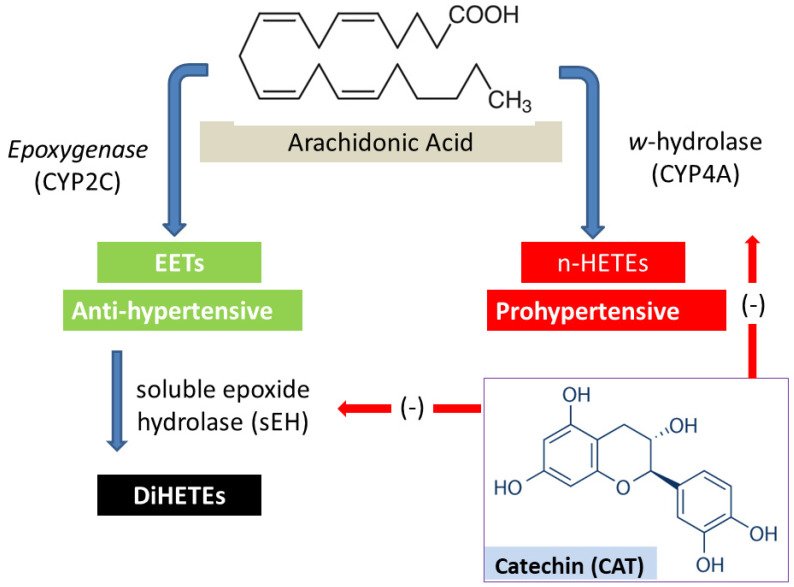
Metabolism of arachidonic acid in the kidney by cytochrome P450 (CYP) enzymes generating vasoconstrictor (n-HETEs) and vasodilator (EETs) metabolites. The hypothesis of the current study is that catechin (CAT) inhibits the *w*-hydrolase (CYP4A) or soluble epoxide hydrolase (sEH) enzymes, and therefore, reduces blood pressure in SHR.

Several cohesive cardiac, vascular, and neurohormonal factors work together to maintain and regulate blood pressure. Evidence from the last two decades indicates that the metabolism of arachidonic acid (AA) by cytochrome P450 (CYP) enzymes in the kidney generates various metabolites that play important and differential roles in the regulation of blood pressure [3,4,5,6]. Extensive evidence supports that Hydroxyeicosatetraenoic acids (HETEs, particularly 20-HETE) and epoxyeicosatetraenoic acids (EETs, Figure 1) are the AA metabolites that play the most significant role in regulating vascular tone and blood pressure [7].

Multiple studies have shown the complex role that 20-HETE plays in blood pressure regulation through the inhibition of sodium reabsorption and enhancing natriuresis, as well as its vasopressor effect on the renal vasculature [6]. A plethora of information has indicated a strong correlation between the rate of formation of 20-HETE in the rat kidney and the kidney expression of CYP4A isoforms, especially CYP4A1, CYP4A2, and CYP4A3 [8]. Our laboratory and others have demonstrated that the inhibition of 20-HETE formation reduces blood pressure in SHR or at least resists the progressive rise in blood pressure in this animal model [7,9,10]. These findings indicate that 20-HETE plays at least a partial role in the pathogenesis of hypertension in SHR.

Epoxyeicosatetraenoic acids (EETs), on the other hand, are identified as endothelium-derived hyperpolarizing factors (EDHF) with diverse biological activities, including renal vasodilation and antihypertensive effects [9]. However, EETs are rapidly degraded into inactive diols by the enzyme soluble epoxide hydrolase (sEH) [9] (Figure 1). The upregulation of sEH and/or reduced availability of EETs are common predisposing factors for blood pressure elevation in SHR [8]. Recently, the inhibition of sEH and increased bioavailability of EETs have been proposed as promising approaches for the prevention and treatment of hypertension. Our research and others have shown that the sub-chronic inhibition of sEH for 1–6 weeks lowers blood pressure and ameliorates the organ damage associated with hypertension [7,10,11,12].

Investigating the effect of naturally occurring phytochemicals on the renal metabolism of AA and their potential to reduce blood pressure has been the focus of our laboratory in the last decade. Our research has identified the blood pressure-lowering effects of several small natural products through the modulation of one or more of the AA metabolic pathways in the kidney [7,12,13]. Catechin (CAT), in Figure 1, is a natural flavonoid that is abundant in plant-based foods, especially cocoa. A substantial body of evidence demonstrates a wide spectrum of the therapeutic benefits of catechin, including cardioprotection and chemopreventive effects [14,15]. The study was undertaken to investigate the antihypertensive effect of CAT in young male spontaneously hypertensive rats following sub-chronic oral administration and to assess the effect of CAT administration on the metabolism of arachidonic acid in the kidney, with a particular focus on sEH and CYP4A. The effect of CAT on blood pressure and AA metabolism in the rat kidney will be compared to captopril (CAP): a potent angiotensin converting enzyme inhibitor (ACEi) and a commonly used anti-hypertensive drug.

## 2. Results

**Figure 2:**
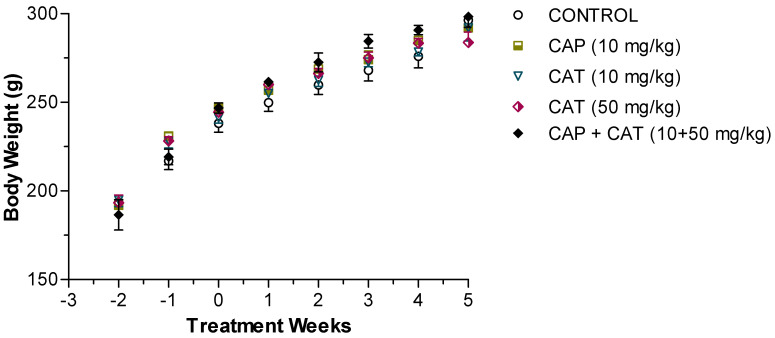
Age-related changes in body weight of male spontaneously hypertensive rats treated with vehicle (control), captopril (CAP), and catechin (CAT). Each point represents the mean ± SEM of six–nine animals.

The oral administration of CAT or CAP for five weeks did not cause any observable changes in SHR behavior, as reported by daily inspections. Figure 2 illustrates the average weekly body weights of SHR in the five study groups. All groups grew normally during the study, and no significant difference in body weight was gained between the CAT- or CAP-treated rats compared to the untreated rats (*p* = 0.981). Similarly, the assessment of the daily consumption of water did not reveal any significant differences among the study groups (19.5 ± 1.3 mL/100 g of body weight, *p* > 0.05).

### 2.1. Effect of Catchin and Captopril Treatment on Blood Pressure

**Figure 3:**
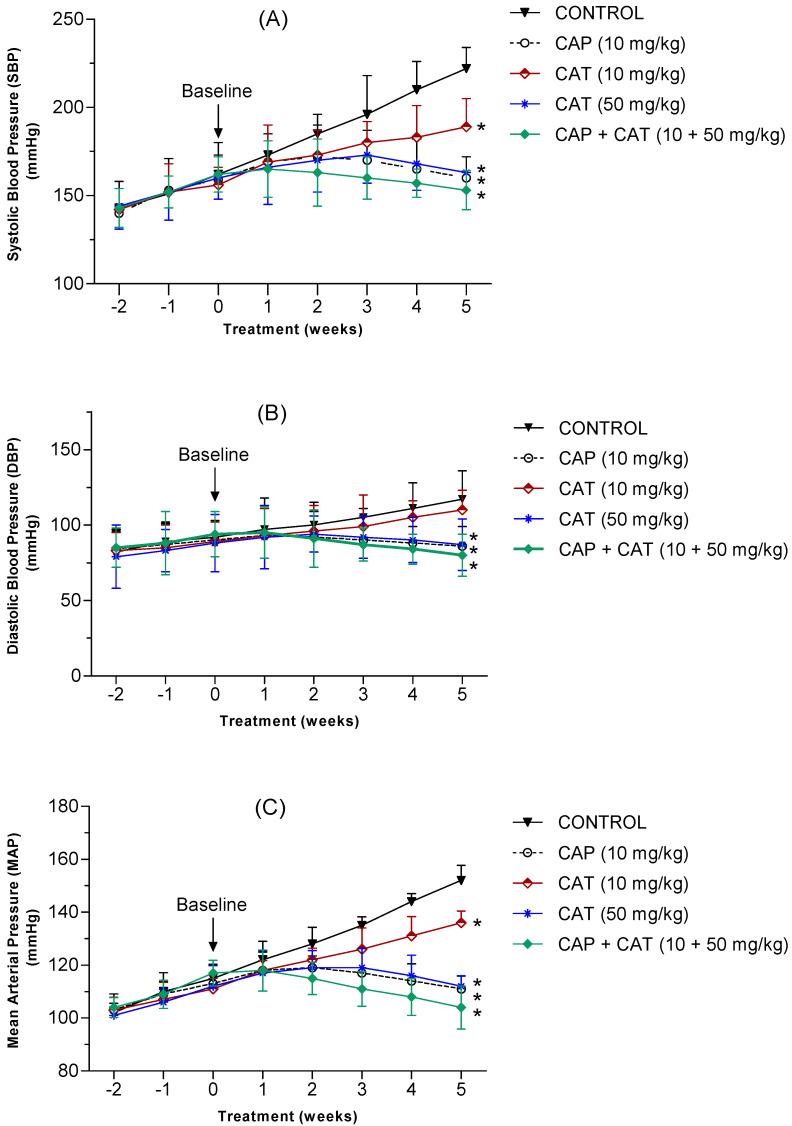
Effect of catechin (CAT) and captopril (CAP) on the systolic (**A**), diastolic (**B**), and mean arterial blood pressure (**C**) in male spontaneously hypertensive rats (SHR). Blood pressure was measured weekly for 5 weeks as described in the methods section. Data are presented as the mean ± SEM (*n* = 6–9). * *Significant difference from control group at 5 weeks, p < 0.05*.

The systolic (SBP), diastolic (DBP), and mean arterial (MAP) blood pressure was measured every 7 days for five weeks to investigate the blood pressure-lowering effect of the daily administration of low and high dose CAT compared to captopril (CAP). Throughout the 2-week acclimation period, blood pressure showed a steady rise in all study groups, with no significant differences among the groups. At the baseline (week zero), no significant difference in SBP, DBP, or MAP was detected between any of the study groups (*p* > 0.05). Untreated control rats demonstrated a steady increase in their blood pressure which was expected in this rat model of hypertension (Figure 3). Specifically, SBP showed a change of +36% in the control group at the end of the study compared to a change of +21% and 1.9% in the low and high CAT doses, respectively (Figure 3A). Captopril monotherapy, on the other hand, resulted in an increase in SBP by only 0.6% after treatment for 5 weeks. Treatment with CAP or high-dose CAT for 5 weeks normalized the rise in SBP as the 5-week SBP readings were not significantly different from the baseline SBP values (*p* < 0.05). Compared to the control group after 5 weeks of administration, all treatment groups had a significantly lower SBP (*p* < 0.05).

Additionally, while DBP increased by +27% in the untreated control rats, a 5-week treatment with low and high CAT doses resulted in a change of +20% and −1.6%, respectively (Figure 3B). Captopril monotherapy, on the other hand, resulted in a change in DBP by −4.4%. Treatment with CAP or high dose CAT for 5 weeks normalized the rise in DBP as the 5-week readings were not significantly different from their baseline DBP values (*p* > 0.05). Compared to the control group after 5 weeks of administration, DBP in the low-dose CAT group was not statistically significantly different from the control group (*p* > 0.05, CI −58 to −4.0). Monotherapy with a high-dose of CAT, normal dose of CAP, or combination of both for 5 weeks resulted in a significant reduction in DBP compared to the control group (*p* < 0.05).

Similarly, While MAP increased by +32% in the untreated control group, treatment with low and high CAT doses for 5 weeks resulted in a change of only +22% and 0.00%, respectively (Figure 3C). Captopril monotherapy, on the other hand, resulted in a reduction in MAP by 18.6%. Treatment with CAP or high-dose CAT for 5 weeks normalized the rise in MAP as the 5-week readings were not significantly different from the baseline MAP values (*p* > 0.05). Compared to the control group, after 5 weeks of administration, all the treatment groups had a significantly lower MAP (*p* < 0.05).

The concomitant administration of CAP and high-dose CAT resulted in a significant reduction in SBP, DBP, and MAP by 5.5%, 15%, and 11%, respectively, compared to the baseline. Compared to the control group, the combination of CAP and high-dose CAT significantly lowered SBP (*p* < 0.0001, CI −96 to −42), DBP (*p* < 0.001, CI −64 to −9.9), and MAP (*p* < 0.001, CI −74 to −22).

### 2.2. Effect of CAP and CAT Treatment on 20-HETE Formation in Renal Microsomes

**Figure 4:**
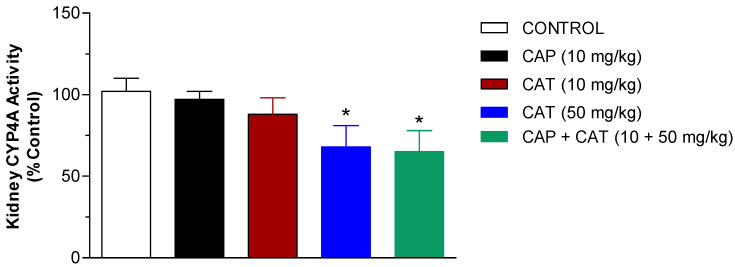
Effect of five week-daily oral treatment of SHR with catechin (CAT) and captopril (CAP) on CYP4A activity measured as 20-HETE formation rate. Data are presented as the mean ± SEM (*n* = 6–9). * *Significant difference from control group at 5 weeks, p < 0.05*.

Extensive research over the last two decades has demonstrated the important role that CYP metabolites of the arachidonic acid in the kidney play in the regulation of blood pressure through their effect on vascular tone and water/salt balance [4]. 20-HETE is a potent vasoconstrictor and a CYP4A-mediated metabolite of AA. We have previously demonstrated the blood-pressure-lowering effect of several small phytochemicals that have a moderate to strong inhibitory effect on renal CYP4A activity [7,14,16]. To inspect the effect of low and high-dose CAT as well as CAP on the activity of CYP4A, we measured the rate of 20-HETE formation in renal microsomes of SHR following daily oral administration for 5 weeks. We analyzed 20-HETE in renal microsomes using a validated LC-MS/MS method, as described in the Methods section. Figure 4 demonstrates that monotherapy with CAP or CAT at 10 mg/kg doses did not have any significant effect on the rate of 20-HETE formation in the rat kidneys. Treatment with a 50 mg/kg dose of CAT resulted in significant inhibition of renal CYP4A activity, as reflected by more than a 30% reduction in the 20-HETE formation rate (*p* < 0.05). The addition of CAP 10 mg/kg dose to the high-dose CAT group did not result in the further inhibition of CYP4A activity, but the 20-HETE formation rate was still significantly lower than the control group (*p* < 0.05) (Figure 4).

### 2.3. Effect of CAP and CAT Treatment on Soluble Epoxide Hydrolase (sEH) Activity

**Figure 5:**
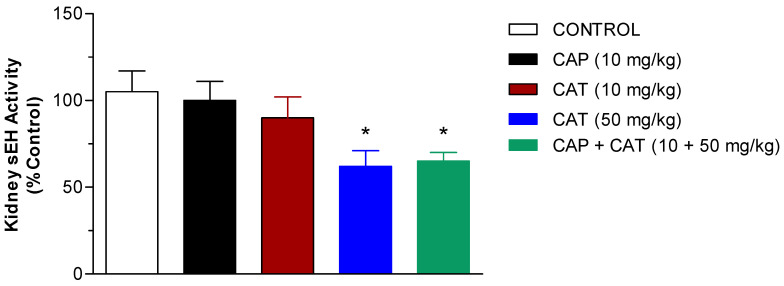
Effect of five week-daily oral treatment of SHR with catechin (CAT) and captopril (CAP) on soluble epoxide hydrolase (sEH) activity in rat kidney cytosols. Data are presented as the mean ± SEM (*n* = 6–9). * *Significant difference from control group at 5 weeks, p < 0.05*.

The inhibition of sEH, the enzyme that degrades the cardioprotective EETs, has demonstrated antihypertensive and renal vascular protective effects [17]. Therefore, we investigated the potential inhibitory effect of CAP and CAT on the activity of this enzyme in kidney cytosolic fractions from the control and CAP- or CAT-treated SHR. To achieve this goal, we used Epoxy fluor 7 as a sensitive fluorescent substrate for sEH, as explained in the methods section. Our findings presented in Figure 5 indicate that monotherapy with CAP or CAT at 10 mg/kg doses did not have any significant effect on the activity of sEH in the rat kidneys. Treatment with a 50 mg/kg dose of CAT resulted in a 40% inhibition of renal sEH activity compared to the control (*p* < 0.05). The co-administration of the CAP 10 mg/kg dose and the high dose CAT did not result in further inhibition in sEH activity but was still significantly lower than the control group (*p* < 0.05), Figure 5.

### 2.4. Correlation between Blood-Pressure Lowering Effect and Enzymatic Activity

**Figure 6:**
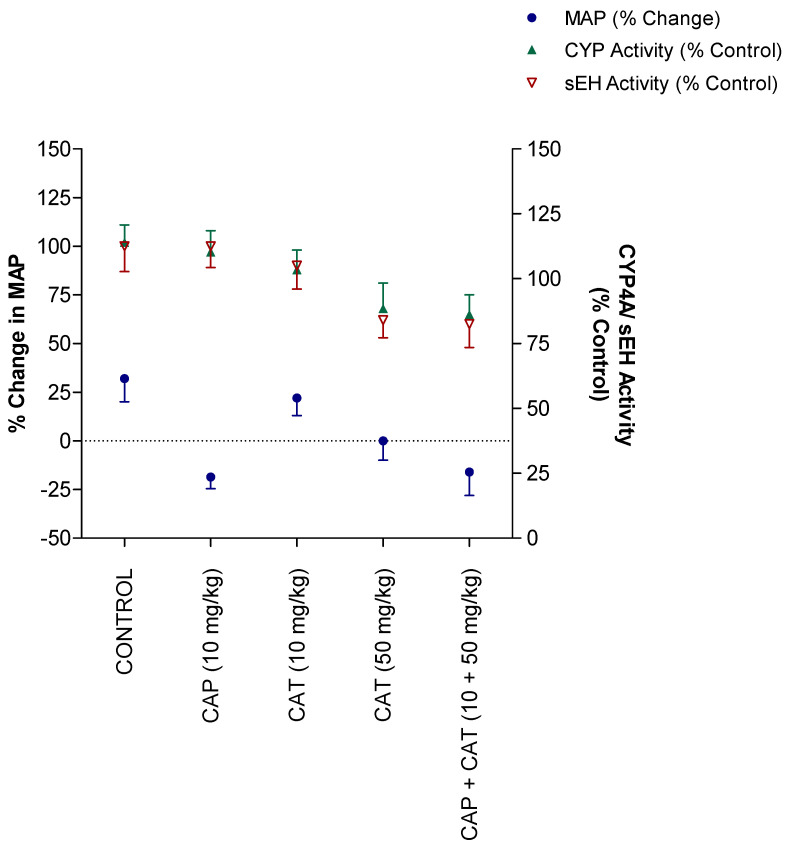
The % changes in mean arterial blood pressure (MAP) and enzymatic activity of CYP4A and sEH activity as a function of different doses of catechin (CAT) and captopril (CAP) in spontaneously hypertensive rats (SHR). Treatment rats received CAP and/or CAT in their drinking water at different concentrations for 5 weeks. Control rats received drinking water only.

To investigate if the inhibitory effect of CAT on the enzymatic activity of CYP4A or sEH contributed to the blood pressure-lowering effect in SHR, we examined the correlation between the different CAT and CAP doses and the magnitude of changes in MAP, CYP4A activity, and sEH activity. The data presented in Figure 6 demonstrate that the changes in MAP induced by CAP monotherapy did not correlate with the changes in CYP4A or sEH activities (*p* > 0.05). On the other hand, the changes in MAP in the CAT-treated groups was significantly correlated with the reduction in CYP4A activity (Pearson r; −0.97 and *p*; 0.025). Specifically, while the 10 mg/kg and 50 mg/kg doses of CAT lowered MAP by between approximately 11 and 26%, CYP4A activity was inhibited by 12 and 32%, respectively. Likewise, the CAT-mediated lowering in MAP were significantly correlated (Pearson r; −0.85 and *p*; 0.04) with the degree of inhibition in sEH activity. Namely, while the 10 mg/kg and 50 mg/kg doses of CAT lowered MAP by approximately 11 and 26%, sEH activity was reduced by 10 and 38%, respectively (Figure 6). Compared to monotherapy with high dose CAT (50 mg/kg), the addition of CAP (10 mg/kg) did not improve the correlation of MAP reduction with either CYP4A or sEH activity despite a more pronounced reduction in MAP.

## 3. Discussion

To our knowledge, this is the first study that examines the effect of the sub-chronic administration of CAT on the blood pressure (BP) of SHR during their peripubertal age when BP undergoes a progressive rise [18]. Our study did not only demonstrate a BP-lowering effect of CAT compared to a well-known antihypertensive medication but also indicated that this effect was at least partially due to CAT-mediated effects on the metabolism of arachidonic acid in the kidney.

Our laboratory has been interested in the past two decades in understanding the health benefits of small natural molecules [7,12,13,16,19,20]. Catechin (CAT) is among the most abundant and commonly studied flavonoid that exists in a wide variety of foods and herbs. Several epidemiological studies, in vitro and in vivo experiments, as well as human clinical studies, have reported the cardiovascular protective effect of CAT or CAT-rich foods such as cocoa and dark chocolate [18,19,21]. Unfortunately, these reports were derived from acute and short-term exposure to CAT and did not offer a mechanism for its health benefits.

The effectiveness of the five-week treatment with CAT in resisting (low dose) and lowering (high dose) the expected steady rise in blood pressure in SHR (Figure 3) was comparable to the blood pressure-lowering effect of Captopril: a commonly used angiotensin-converting enzyme inhibitor, that was implemented in this study as a positive control. Similarly, the effect of CAT in reducing blood pressure was very comparable to commonly used anti-hypertensive medications that act by different mechanisms. Specifically, amlodipine (a calcium-channel blocker), telmisartan (an angiotensin receptor blocker), hydrochlorothiazide (a diuretic), and lisinopril (an angiotensin-converting enzyme inhibitor) resulted in a comparable MAP reduction of 15%, 17%, 18%, and 20%, respectively [21,22,23].

A large body of evidence in the last two decades indicated that the CYP-mediated metabolism of arachidonic acid (AA) in the kidney generates metabolites that play a key role in blood pressure regulation, namely, 20-HETE and EETs [24,25]. The present study shows that the sub-chronic exposure of SHR to low CAT doses reduces the progressive rise in blood pressure that was seen in the control untreated rats. Exposure to a high CAT dose, on the other hand, reduced blood pressure after 5 weeks of daily administration. This effect on blood pressure by CAT was accompanied by a comparable inhibitory effect on the activity of renal CYP4A: the enzyme responsible for the formation of the vasoconstrictor metabolite, 20-HETE (Figure 4). Our findings support earlier reports that the upregulation of the formation of 20-HETE contributes to the elevation in oxidative stress, endothelial dysfunction, and the increase in peripheral vascular resistance associated with elevated blood pressure [26,27,28]. Similarly, the inhibition of 20-HETE production following the administration of CYP4A1/2 inhibitors [29] or CYP4A1 antisense oligonucleotide [26] has demonstrated reduced blood pressure and vascular tone in SHR. The administration of Captopril, on the other hand, resulted in the expected reduction in blood pressure but without significant changes in 20-HETE formation (Figure 4). Although the coadministration of CAP and high-dose CAT resulted in a synergistic blood-pressure-lowering effect (Figure 3), the effect on the 20-HETE formation rate (i.e., CYP4A activity) was not statically significantly different from high-dose CAT alone (Figure 4). Our data indicate that the inhibition of 20-HETE formation might be a contributing factor to the observed blood pressure-lowering effect of CAP. However, the antihypertensive effect of CAP is not mediated by the modulation of the CYP4A-mediated metabolism of arachidonic acid.

There is ample evidence to support the hypothesis that the pathogenesis of hypertension in SHR is largely due to the rapid degradation of the vasodilator AA metabolites and EETs, to their corresponding and biologically inactive diols, DiHETEs [11,30]. Therefore, the inhibition of sEH and enhancing the bioavailability of EETs have been suggested as a relevant therapeutic strategy for the prevention of hypertension and protecting the kidney from hypertension-induced damage [11,17]. Earlier studies have demonstrated that the acute and chronic inhibition of sEH activity increases EET levels, lowers arterial blood pressure, and perfects against end-organ damage associated with renal and cardiovascular diseases [7,12,14,20]. The current study indicates that the high dose of CAT reduces the renal activity of sEH by 40% (Figure 5), and this inhibition was significantly correlated to lowering MAP (Figure 6). Monotherapy with CAP did not cause any significant effect on the activity of the sEH enzyme. Additionally, the coadministration of CAP and high doses of CAT did not cause the inhibition of the enzyme compared to high doses of CAT alone. While our data may indicate that the blood-pressure-lowering effect of CAP is not due to the modulation of the activity of sEH, the CAT-mediated inhibition of this enzyme is at least contributing to its effect on blood pressure. The strategy of targeting sEH as a novel approach for treating cardiovascular and renal diseases was introduced in the early 2000s. Despite the potent antihypertensive effect of the synthetic sEH inhibitors (such as chalcone oxide, glycosidol, carbamates, and urea derivatives), the undesirable physicochemical and/or pharmacokinetic properties significantly hindered their therapeutic use [27,28]. Our laboratory has been interested in developing natural sEH inhibitors as an attractive target for lowering blood pressure with minimal risk of adverse effects and drug–drug interactions since sEH is not primarily involved in drug metabolism.

The novelty of this line of research lies in identifying CYP4A and soluble epoxide hydrolase as potential targets to lower blood pressure. We have used a series of docking and in vitro studies to screen our bank of small natural products (isothiocyanates and flavonoids) and to test their potential to inhibit one or both of these enzymes before testing the in vivo effect of the most potent inhibitors. Out of the 27 tested compounds, we have identified four, including catechin. Additionally, in this study, we have included captopril as a positive control blood pressure-lowering drug and as a monotherapy and add-on to catechin to (1) compare the efficacy of catechin in this commonly used antihypertensive medication and (2) investigate the utility of catechin as an add-on therapy to captopril, especially for patients who are reluctant to take many medications or cannot afford their high cost.

Our study is not without limitations. Although the data presented in Figure 6 indicate a correlation between CAT-mediated reduction in MAP and the inhibition of CYP4A and sEH activities, it is beyond the conclusion of the study’s findings to solely attribute the antihypertensive effect of CAT to its effect on AA metabolism. It also should be noted that other pathways contribute to the metabolism of 20-HETE, including CYP2C, alcohol dehydrogenase, lipoxygenase, cyclooxegenase, glucuronosyltransferase, and β-oxidation pathways [4]. Additionally, the antioxidant effect of CAT in SHR may play a role in its antihypertensive effect [15]. These findings indicate that CAT lowers blood pressure in SHR by different mechanisms, and the modulation of AA metabolism in the kidney is one of them. Another limitation of our study is that we did not examine the liver tissues for any possible hepatotoxicity. Earlier reports have associated catechin administration with hepatotoxicity [31]. In fact, most of the studies were derived from large doses of Epigallocatechin-3-gallate (EGCG) in a green tea extract, which may be contaminated by pyrrolizidine alkaloids (PA), and that 1,2-unsaturated PA can be activated by CYP450 enzymes to form hepatotoxic metabolites [32]. Additionally, a recent article demonstrated that EGCG attenuates methotrexate-induced hepatotoxicity through its antioxidant effect [33]. Finally, our rats were examined on a daily basis during the 5-week study, and we did not observe any signs or symptoms of hepatotoxicity, such as changes in animal behavior or urine/eye color.

## 4. Materials and Methods

### 4.1. Materials

Arachidonic acid, 20-HETE, and *d*4-20-HETE (the internal standard) were purchased from the Cayman Chemical Company (Ann Arbor, MI, USA). Catechin, Captopril, and all chemicals used in the enzymatic assays were purchased from Sigma-Aldrich (ST. Louis, MO, USA) and were of the highest grade available. Organic solvents used in the LC-MS/MS analysis were HPLC-grade (Fisher Scientific, Pittsburg, PA, USA).

### 4.2. Animals

All experiments were performed in accordance with the protocols and institutional guidelines established by Pacific University. Five-week-old male SHRs were obtained from Charles River Laboratories (Wilmington, MA, USA) and were housed, two per cage, under a constant temperature (23–25 °C), 12 h dark/light cycle, and fed a standard laboratory chow with water *as libitum*. An acclimation period of 2 weeks for blood pressure measurement and drug administration was allowed before the initiation of the experimental period.

### 4.3. Preparation of Catechin (CAT) and Captopril (CAP) Solutions

All catechin solutions were made fresh daily in water containing 0.5% ethanol at concentrations to provide doses of 10 and 50 mg/kg. Captopril solutions were prepared daily in water containing 0.5% ethanol to provide doses of 10 mg/kg. The combined solution of CAP and CAT was made daily in water containing 0.5% ethanol to provide doses of 10- and 50 mg/kg, respectively. According to our preliminary stability studies of CAT and CAP, no significant degradation of either chemical was detected for up to 5 days.

### 4.4. Catechin (CAT) and Captopril (CAP) Treatment

Thirty-three rats were randomly assigned to one of the 5 experimental groups (*n* = 6–9). Group 1 served as the negative control group and received 0.5% ethanol in drinking. Group 2 served as a positive control group and received CAP at a dose of 10 mg/kg in drinking water daily for 5 weeks. Groups 3 and 4 served as treatment groups and received CAT at concentrations of 10 and 50 mg/kg, respectively, in their drinking water daily for 5 weeks. Group 5 received a mixture of CAP and CAT at concentrations of 10 and 50 mg/kg, respectively, in their drinking water daily for 5 weeks. Body weight was measured every week directly after measuring blood pressure.

### 4.5. Blood Pressure Measurements

Systolic (SBP), diastolic (DBP), and mean arterial (MAP) blood pressure were measured at 7-day intervals for 5 weeks in conscious, pre-warmed, and restrained rats using the CODA™ tail-cuff blood pressure system, CODA-HT4 (Kent Scientific, Torrington, CT, USA) as described previously [7]. Each reported blood pressure value is the average of at least ten stable measurements made in every session from individual rats.

### 4.6. Tissue Collection

At the end of the 5-week treatment, the animals were exposed to brief CO_2_ anesthesia and subsequently killed by decapitation. The kidneys were rapidly removed, rinsed with ice-cold saline, and snap-frozen in liquid nitrogen, then stored at −80 °C until use. Kidney microsomal and cytosolic fractions were prepared from the renal cortex using an ultracentrifugation protocol as described previously [12].

### 4.7. Quantification of 20-HETE Metabolite in Rat Kidney Microsomes

To investigate the effect of CAP and CAT on CYP4A activity, the 20-HETE formation rate was measured in renal cortical microsomes prepared from the control and CAP- or CAT-treated rats, according to Yue et al. [34], with slight modifications. Briefly, the renal microsomal protein (500 µg) was incubated in a mixture containing arachidonic acid (100 mM), MgCl_2_ (10 mM), and KCl (150 mM) in 1 mL of 100 mM potassium phosphate buffer (pH 7.4). The incubation reaction was initiated by adding NADPH (1 mM) and carried out for 15 min at 37 °C, then terminated with 20 µL of 2N HCL. Arachidonic acid and 20-HETE were extracted thrice with ethyl acetate; the combined organic phase was evaporated under nitrogen, and the dry residue was reconstituted in 400 µL of an acetonitrile/water/formic acid (59.3:40:0.70%) mixture.

### 4.8. Measurement of Soluble Epoxide Hydrolase Activity Using Fluorescence Assay

To examine the effect of CAP and CAT on the sEH activity in kidney cytosol, Epoxy Fluor 7 (Cayman Chemical Co. Ann Arbor, MI, United States) was utilized as a sensitive fluorescent substrate. The formation of the highly fluorescent metabolite was monitored at excitation and emission wavelengths of 330 and 465 nm, respectively, on a Synergy2^®^ microplate reader using Gen5 Software (BioTek Winooski, VT, USA) as described previously [35].

### 4.9. Data Analysis

Data were analyzed using a one-way analysis of variance (ANOVA), followed by Tukey’s post hoc test to detect differences between the groups. A statistical significance was considered with a probability of *p* < 0.05. Data (animal body weight, blood pressure, and enzymatic activity) were presented as the mean ± standard error of the mean (SEM). The correlation between variables was determined using Pearson’s correlation coefficient (r). GraphPad Prism 5.0 (GraphPad Software Inc., San Diego, CA, USA) was used for statistical analysis and graphical representation.

## 5. Conclusions

In conclusion, these data demonstrate that catechin reduces the elevated blood pressure in SHR to a degree comparable to captopril: a commonly used antihypertensive medication. Additionally, this blood pressure-lowering effect of catechin, but not captopril, was considerably correlated with the inhibition of 20-HETE formation and sEH activity in the kidney. Our findings could propose catechin as a cost-effective, stand-alone, or complementary approach for the treatment of hypertension and the prevention of organ damage induced by uncontrolled high blood pressure.

## Data Availability

Not Applicable.
